# Moral emotions in motion: a cross-sectional study of shame, guilt, and disordered eating in UK university athletes

**DOI:** 10.1186/s40337-026-01543-8

**Published:** 2026-02-13

**Authors:** Larysa Zasiekina, Molly Dunn, Lara Miller, Imogen Tattersall, Victoria Vidaurre

**Affiliations:** 1 Department of Psychology, University of Exeter, Washington Singer Building, Perry Road, Exeter, EX4 4QG UK; 2 Department of General and Clinical Psychology, Lesya Ukrainka Volyn National University, 13 Voli Avenue, Lutsk, 43025 Ukraine

**Keywords:** Eating pathology symptoms, Nonelite athletes, Weight-sensitive sports, Moral emotions, Shame, Guilt

## Abstract

**Background:**

This research focuses on subclinical eating pathology symptoms (EPS) in young adult university athletes, addressing a gap in studies involving nonelite athletes. Social transitions, athletic and academic pressures, body shame, and participation in weight-sensitive sports contribute to an elevated risk of EPS. This study aims to examine how demographic factors (gender, age, type of sport, living arrangements, and duration of membership) and adverse moral emotions (shame and guilt) predict EPS. Additionally, it investigates whether shame and guilt mediate the relationship between sport type and EPS.

**Methods:**

Participants (*N* = 130), excluding individuals with clinical eating disorders, completed validated self-report measures: the Eating Pathology Symptoms Inventory and the State Shame and Guilt Scale (SSGS). Ethical approval was granted by the Department of Psychology Research Ethics Committee at the University of Exeter (Reference: 8485441). The study was prospectively registered on the OSF prior to data collection (10.17605/OSF.IO/5R3T8).

**Results:**

Hierarchical regression analysis revealed that participation in weight-sensitive sports and higher scores on the SSGS were significant positive predictors of EPS. Mediation analysis indicated no significant indirect effect of shame or guilt on the relationship between sport type and EPS.

**Conclusion:**

These findings suggest that involvement in weight-sensitive sports and high levels of shame and guilt are key risk factors for EPS in young adult athletes. Conversely, participation in non-weight-sensitive sports appears to serve as a protective factor. This study underscores the urgent need for targeted interventions and increased awareness of subclinical eating pathology in this population.

## Abstract (plain language summary)

This research investigated eating difficulties in young adult athletes at UK universities, specifically those who play sports but are not elite or professional athletes. Many university students face major life changes, such as moving away from home and managing academic stress, which can affect their relationship with food. Athletes can also feel pressure to look a certain way or perform well in their sport. These pressures may lead to unhealthy eating habits, even if individuals do not have a diagnosed eating disorder.

The study focused on eating pathology symptoms (EPS), which include unhealthy eating behaviors such as skipping meals, overeating, or exercising in a harmful way. Researchers have wanted to understand what factors might predict these behaviors in university athletes. They looked at personal characteristics such as gender, age, type of sport played, living situation, and how long someone had been involved in their sport. They also investigated how emotions such as shame and guilt might be linked to eating problems.

A total of 130 student-athletes without a clinical diagnosis of an eating disorder completed surveys that measured their eating behaviors and feelings of shame and guilt. The results revealed that athletes who played weight-sensitive sports, such as gymnastics or long-distance running, were more likely to have unhealthy eating behaviors. High levels of shame or guilt were also linked to increased EPS. However, shame and guilt did not explain the connection between the type of sport and EPS.

In summary, playing sports where weight or body shape matters and experiencing intense shame or guilt puts young athletes at greater risk for eating problems. This study shows how important it is for universities to create support systems that help student athletes manage these pressures and reduce their risk of developing more serious eating disorders.

## Background

In the UK, an estimated 1.25 million people live with eating disorders, with the majority of cases occurring in individuals under the age of 25 [14, 28]. Eating disorder pathology encompasses a continuum of disordered eating behaviors, such as restrictive eating, bingeing, and compulsive exercise, that significantly affect psychological and physical well-being [20]. These behaviors are associated with serious health consequences, including cardiovascular complications, nutritional deficiencies, and bone density loss [17], in addition to heightened risks of depression, anxiety, and chronic mental health problems [42].

Eating pathology symptoms (EPS), the empirical manifestation of eating disorder pathology, range from subclinical issues to diagnosable disorders [48]. While the DSM-5 defines clinical criteria for eating disorders, subclinical behaviors, although not meeting diagnostic thresholds, still present serious risks [24, 47]. Moreover, nearly half of the general population reports problematic thoughts or behaviors related to food, body image, or exercise [8], highlighting the importance of examining EPS in nonclinical populations.

Although research has often focused on adolescents, emerging adulthood is increasingly recognized as a critical period for the onset of eating disorder pathology [19, 33]. University students, particularly first-year university students, experience disruptions in their eating routines, increased autonomy, and academic stress, which can heighten their vulnerability to maladaptive coping strategies [11, 39, 40, 46]. Within this population, athletes represent a high-risk group. Despite the physical and psychological benefits of sport participation, athletes face distinct pressures surrounding body image and performance [4, 25]. Regular training and competition can exacerbate disordered behaviors, particularly in weight-sensitive sports such as gymnastics, cheerleading, or boxing [2, 10]. In particular, female athletes are disproportionately affected by the societal ideals of thinness and aesthetic expectations. As a result, behaviors such as overtraining, dehydration, and rapid weight loss are often reported [7, 16, 38, 44]. Paradoxically, despite being physically fit, athletes often report high levels of body dissatisfaction and shame [9, 37].

Emerging evidence suggests that moral emotions, especially shame and guilt, may play a crucial role in the development of eating disorder pathology in athletes. Shame, as a negative self-evaluation, is linked to perceived failure to meet internalized ideals of appearance or performance [26, 32, 50], whereas guilt is related to specific behaviors and may lead to compensatory actions such as over-exercising [6]. Although both emotions are relevant, shame has shown stronger and more consistent associations with eating disorder pathology than guilt does [5, 32].

Despite a growing body of literature, much of the recent research has focused on elite athletes or adolescent samples, leaving nonelite university athletes underexplored. Given that more than two-thirds of university students in the UK participate in sport societies, there is a pressing need to examine disordered eating patterns in this accessible yet vulnerable group.

This cross-sectional study investigated subclinical EPS among young adult athletes in UK university sports clubs. This study aims to identify key predictors of EPS, including demographic characteristics and moral emotions of shame and guilt, and explore the psychological mechanisms linking sport-specific pressures to disordered eating.

The study addresses three research questions:

- RQ1: To what extent do demographic factors (age, gender, sport type, living arrangements, and club participation length), alongside shame and guilt, predict EPS in young adult athletes?
- RQ2: Do shame and guilt mediate the relationship between sport type (weight-sensitive vs. non-weight-sensitive) and EPS?
- RQ3: Which subscales of EPS are most strongly correlated with the total score of the State Shame and Guilt Scale (SSGS)?

By addressing these questions, this study contributes to the development of evidence-based prevention and intervention strategies specifically tailored to the needs of university athletic populations.

## Methods

### Participants

From an initial sample of 154 participants, 24 were excluded because of a self-reported clinical eating disorder diagnosis, nonmembership in a university sports society, or incomplete survey responses. This resulted in a final sample of 130 young adult athletes (mean age = 20.25 years, SD = 1.29), all of whom were active members of a sports society at the University of Exeter. Of these, 58.5% were female, and 39.2% were male.

The inclusion criteria were as follows: had a current membership in the University of Exeter Sports Society, was aged between 18 and 25 years, and had no personal history of diagnosed eating disorders or related mental health conditions. The exclusion of individuals with eating disorders was undertaken to ensure methodological consistency and was not intended to discriminate against any protected characteristics as defined by the Equality Act 2010.

Participants were recruited online through the University of Exeter Athletic Union. The Athletic Union President disseminated the study advertisement to 52 club captains, who were asked to circulate it among their members. Some reluctance to participate in the study may have been influenced by barriers such as academic and athletic time constraints, limited perceived incentives, or a lack of interest in the study topic. The participants were invited to enter a random prize draw for a chance to win Amazon vouchers as a token of appreciation. Power analysis indicates that for hierarchical regression with six predictors, a sample size of 133 participants is sufficient to detect medium effect sizes (f² = 0.15), with 80% power at an alpha level of 0.05. For the mediation analysis, assuming medium effect sizes for both the a-path (type of sport → shame/guilt) and b-path (shame/guilt → EPS), approximately 70–80 participants are required to achieve 80% power.

This study employed a quantitative, cross-sectional design to investigate the relationships between demographic factors, moral emotions of shame and guilt, and EPS among young adult athletes. Data were collected via an online survey hosted on Qualtrics, which participants completed at their convenience. The study was prospectively registered on the OSF prior to data collection (10.17605/OSF.IO/5R3T8).

Data collection took place between February and March 2025. The participants completed a self-report survey that included questions on sex, age, living arrangements, sport society membership, and duration of involvement. They then completed two standardized psychological scales: the Eating Pathology Symptoms Inventory and the State Shame and Guilt Scale [22]. All university sports societies were classified as either weight sensitive or nonweight sensitive on the basis of established criteria [2].

The diagnostic assessment tools included the following measures.

The Eating Pathology Symptoms Inventory (EPSI) [12] is a 45-item self-report scale assessing a broad spectrum of subclinical and clinical EPS across eight subscales: binge eating, body dissatisfaction, cognitive restraint, excessive exercise, muscle building, negative attitudes toward obesity, purging, and restricting. The items are rated on a 5-point Likert scale (0 = “Never” to 5 = “Very often”), with higher scores indicating greater symptom severity. The EPSI has demonstrated strong psychometric properties, including high internal consistency (Cronbach’s α > 0.80 across all subscales) and test-retest reliability [30].

The State Shame and Guilt Scale (SSGS) is a 10-item self-report instrument measuring momentary feelings of shame and guilt. The items are rated on a 5-point Likert scale (1 = “Not feeling this way at all” to 5 = “Feeling this way strongly”). The scale includes two subscales, Shame and Guilt, with scores ranging from 5 to 25 for each. Higher scores reflect stronger emotional experiences. The SSGS has demonstrated good internal reliability (Cronbach’s α: Shame = 0.89; Guilt = 0.80) and has been validated in nonclinical samples, making it suitable for research with student-athlete populations [1].

To address the first research question, a hierarchical multiple regression analysis was conducted to examine the predictive value of demographic factors (age, gender, type of sport, living arrangements, and membership duration) and personal variables (shame and guilt) on EPS. The assumptions of regression, including linearity, multicollinearity, homoscedasticity, and normality of residuals, are tested. In step 1, the demographic variables were entered into the model to assess their initial predictive power. In step 2, scores from the SSGS were added to determine whether shame and guilt significantly predicted EPS while controlling for demographic factors.

**Fig. 1:**
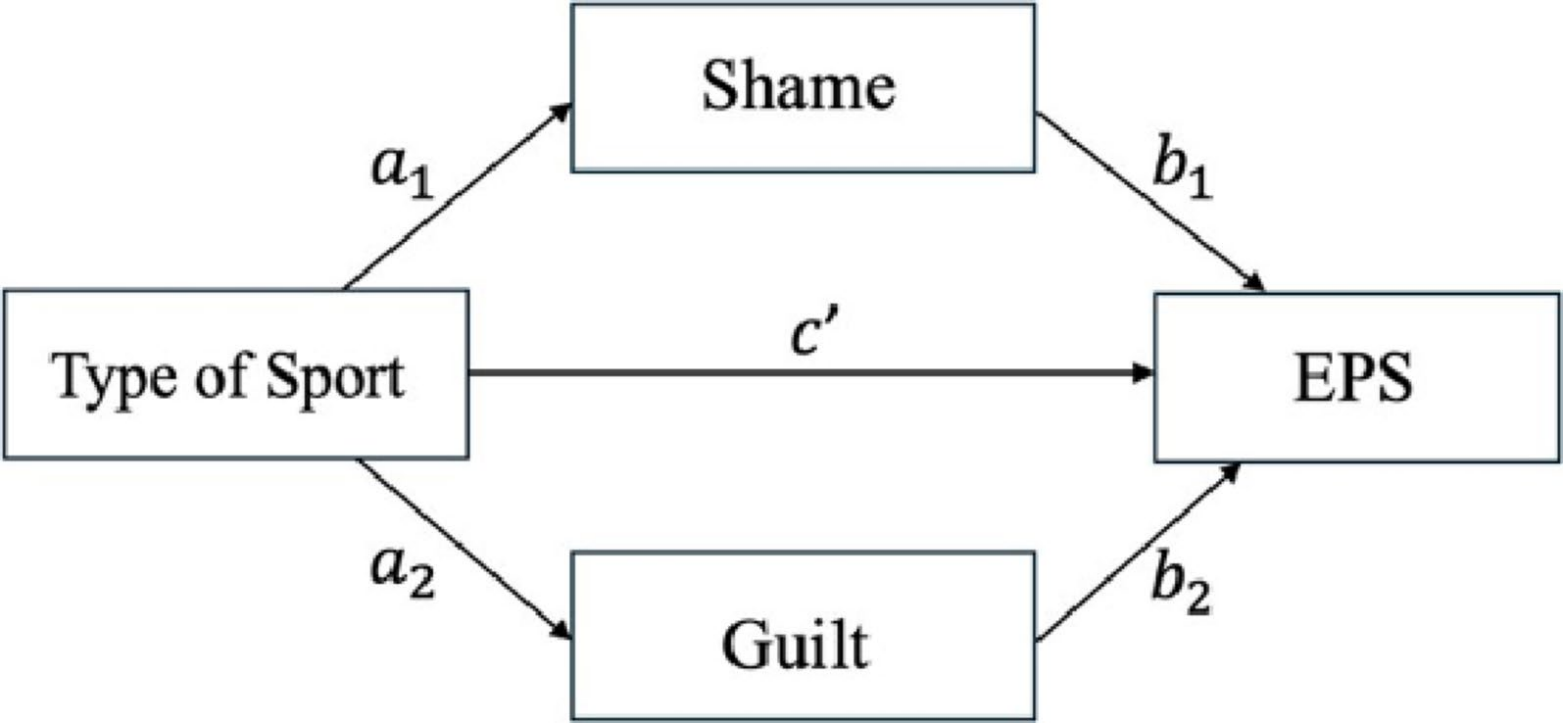
Planned mediation model of the effects of shame and guilt on the relationship between the type of sport and EPS. *EPS* Eating pathology symptoms

To address the second research question, a mediation analysis was conducted via Hayes’ PROCESS macro [15] to examine whether shame and guilt mediated the relationship between the type of sport and EPS. Specifically, the analysis assessed both the direct effect of type of sport (weight-sensitive vs. non-weight-sensitive) on EPS and the indirect effects through the proposed mediators. The following pathways were tested: (a) the direct pathway from the type of sport to EPS and (b) the indirect pathways whereby the type of sport influences the level of shame or guilt, which in turn affects EPS. See Fig. 1 for the planned mediation model.

To address the third research question, a correlational analysis was conducted to examine the associations between self-conscious negative affect and specific dimensions of eating pathology. Pearson correlation coefficients were calculated between the total score of the SSGS and the individual subscales of EPS. Using the total SSGS score allowed us to capture the overall burden of combined shame and guilt, while examining the EPS subscales enabled us to identify the specific symptom domains most strongly associated with these self-conscious emotions. This approach extended our understanding of how shame and guilt relate to different manifestations of eating pathology.

## Results

Table 1 shows descriptive statistics of all the variables in the study.

**Table 1:** Descriptive statistics of independent and dependent variables in the study

	Levels	*N*	Mean (SD)
*Independent variables*			
Gender	Male	51	1.64 (0.58)
	Female	76	
	Other	3	
Age	18	12	4.25 (1.29)
	19	26	
	20	35	
	21	38	
	22	12	
	23	7	
Type of sport	Weight-Sensitive	43	0.67 (0.47)
	Non-Weight-Sensitive	87	
Living arrangement	Alone	1	2.43 (0.67)
	Friends/Roommates	83	
	University Accommodation	36	
	Family	9	
	Other	1	
Membership duration (months)	Less than 6	20	2.94 (1.17)
	6 to 12	25	
	13 to 24	36	
	25 to 36	41	
	More than 36	8	
State shame and guilt scale	Shame	130	7.88 (3.43)
	Guilt	130	9.05 (4.15)
	Total Score	130	16.94 (6.96)
*Dependent Variable*			
Eating pathology symptoms		130	62.47(19.15)

### Hierarchical regression

Multiple hierarchical regression analysis was conducted to examine the predictors of EPS. In Model 1, gender, age, type of sport, living arrangements, and membership duration were entered as predictors. Our results showed that the first model was significant, *F* (5,124) = 3.28, *p* =.008, explaining 11.7% of the variance in EPS (Adj. R² = 0.081). However, after adding the SSGS score in Model 2, the overall model fit improved significantly, *F* (6,123) = 4.95, *p* <.001, increasing the explained variance to 19.5% (Adj. R²= 0.16). The inclusion of SSGS led to a statistically significant ∆*F* (1,123) = 11.88, *p* <.001, *∆R2* = 0.078, demonstrating that SSGS added substantial explanatory power to the model. The full details of these regression models can be found in Table 2.

**Table 2:** Hierarchical regression analysis for variables predicting EPS among young adult athletes

Predictor	B	SE	β	*p*
*Model 1: Demographics*				
*R* ^2^ *∆R* ^2^ F	0.117 0.081 3.277			
Gender	4.741	2.902	0.144	0.105
Age	− 0.071	1.554	− 0.005	0.964
Type of Sport	− 11.498	3.527	− 0.284	0.001**
Living Arrangements	− 2.814	2.771	− 0.098	0.312
Membership Duration	2.271	1.798	0.139	0.209
*Model 2: Personal Variables*				
*R* ^2^ *∆R* ^2^ **F**	0.195 0.155 4.951			
Gender	3.614	2.801	0.110	0.199
Age	0.058	1.490	0.004	0.969
Type of Sport	− 11.437	3.382	− 0.282	< 0.001***
Living Arrangements	− 1.486	2.684	− 0.052	0.581
Membership Duration	2.467	1.725	0.151	0.155
SSGS	0.785	0.228	0.285	< 0.001***

*EPS* Eating pathology symptoms, *SSGS* State Shame and Guilt Scale

**p<.01; ***p<.001

**Fig. 2:**
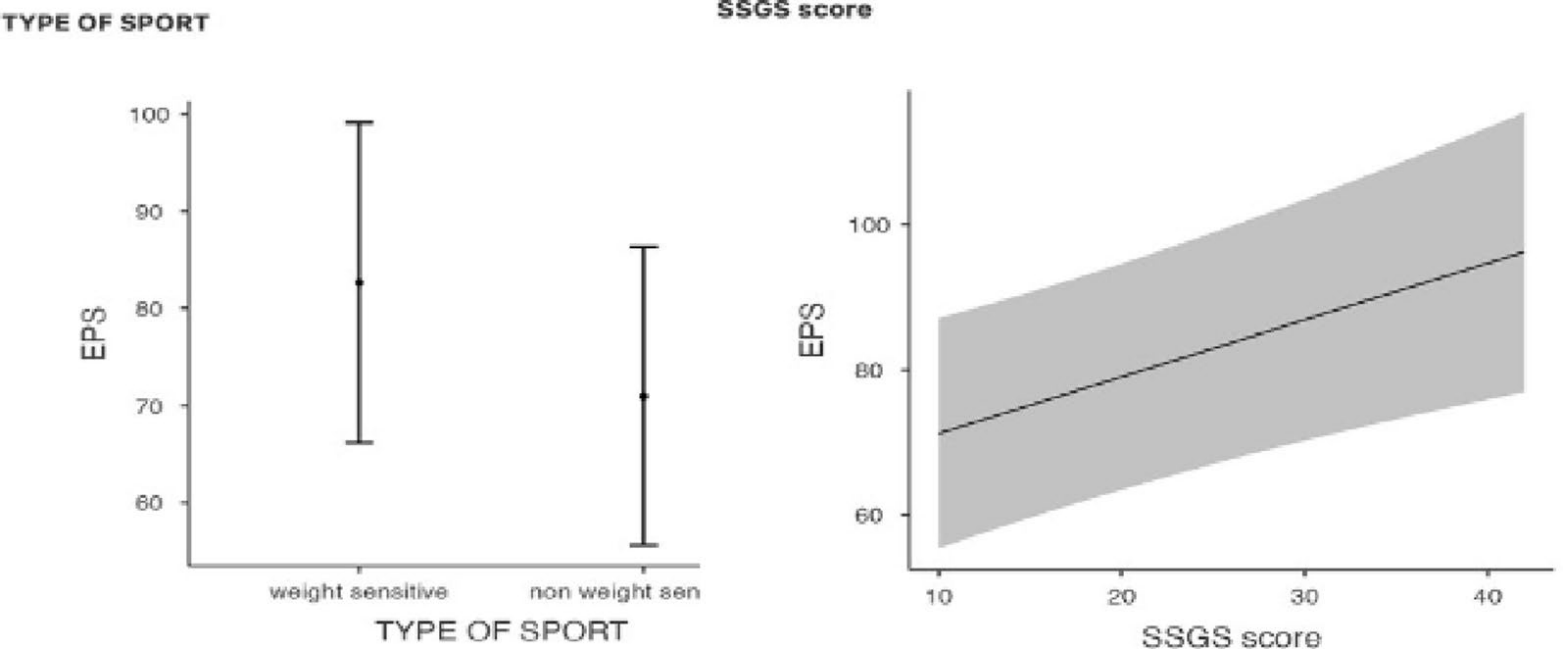
Partial effects of significant predictors (type of sport, SSGS) on EPS. *SSGS* State Shame and Guilt Scale, *EPS* Eating pathology symptoms. The error bars represent the ± standard error. The gray band shows the 95% confidence interval

Figure 2 illustrates the partial effects of the significant predictors of EPS.

**Fig. 3:**
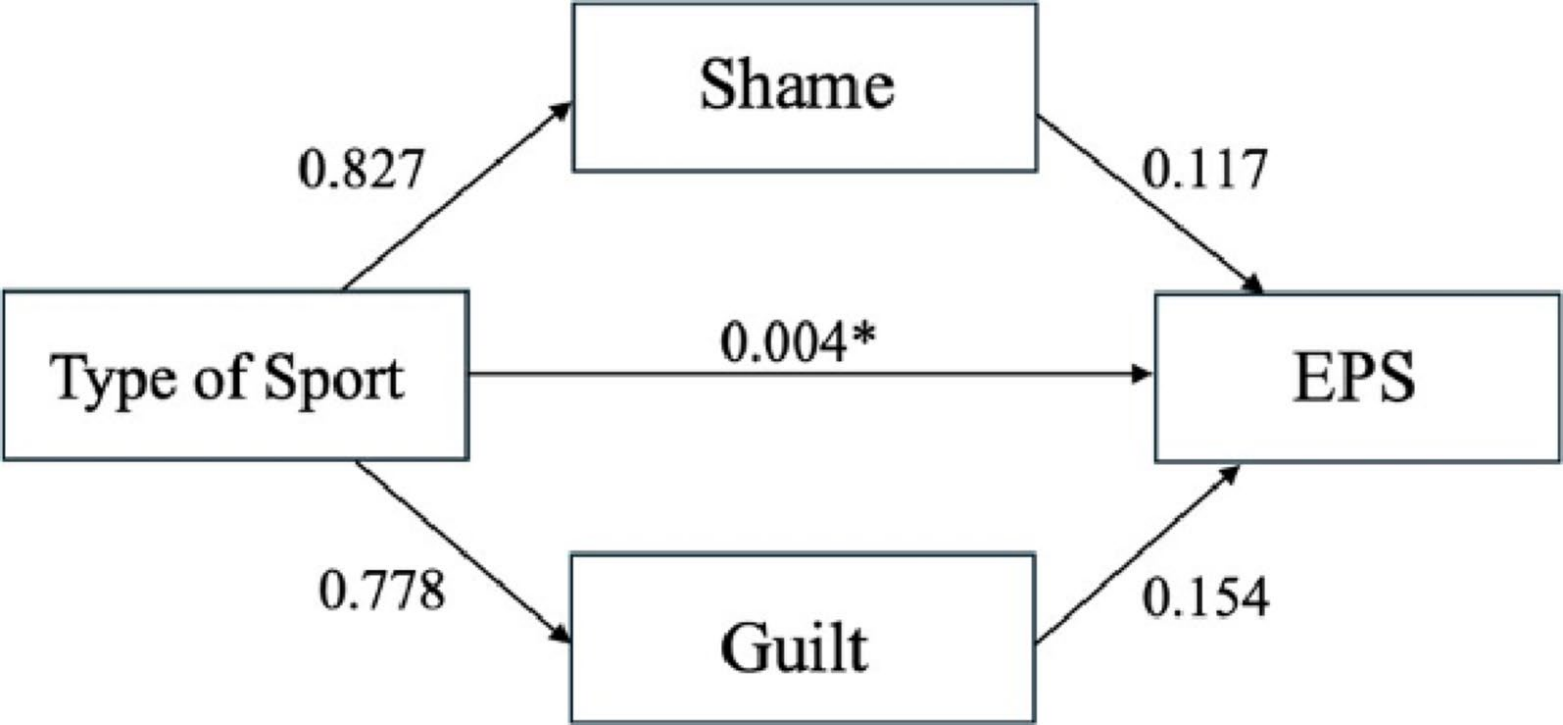
Mediating Effects of Shame and Guilt on the Relationships between the Type of Sport and EPS. *EPS* Eating pathology symptoms

### Mediation effect of shame and guilt

A mediation analysis was conducted to examine whether shame and guilt mediated the relationship between the type of sport and EPS, addressing research question two. The direct effect of the type of sport on EPS remained significant (*b* = −9.852, *SE* = 3.328, *p* =.004, 95% CI [−16.439, − 3.265]), suggesting a negative impact of the type of sport on EPS, independent of the mediators. However, the indirect effects through shame (*b* = 0.139, *SE* = 0.735, 95% CI [**-**1.196, 1.968]) and guilt (*b* = 0.163, *SE* = 0.709, 95% CI [−1.185, 1.823]) were not statistically significant, as their confidence intervals included zero. See Fig. 3 for the mediation model. An examination of the individual paths revealed that the type of sport was not significantly associated with shame (*b* = 0.140, *p* =.827, 95% CI [−1.123, 1.408]), nor was shame significantly associated with EPS (*b* = 0.990, *p* =.117, 95% CI [−0.252, 2.231]). The association between the type of sport and guilt was nonsignificant (*b* = 0.220, *p* =.778, 95% CI [−1.318, 1.757]), as was the relationship between guilt and EPS (*b* = 0.742, *p* =.154, 95% CI [−0.282, 1.766]).

Despite the lack of mediation, the total effect of the type of sport on EPS remained statistically significant (*b* = −9.550, *SE* = 3.483, *p* =.007, 95% CI [−16.442, −2.658]), suggesting that other factors may account for this relationship. These results indicate that shame and guilt do not mediate the effect of the type of sport on EPS; rather, the type of sport directly impacts EPS (see Fig. 3).

**Fig. 4:**
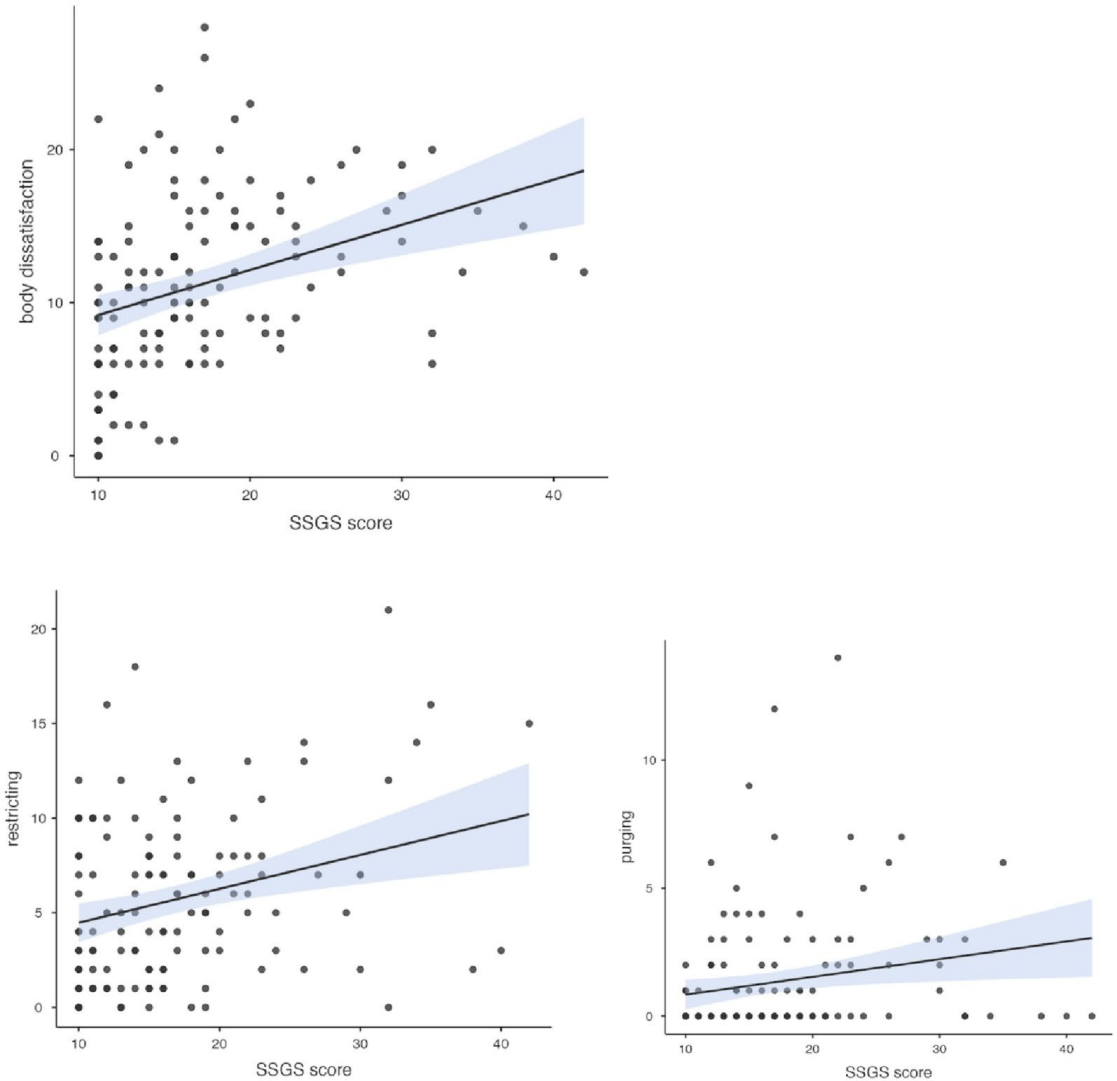
Statistically significant relationships between EPSI subscales (body dissatisfaction, purging, and restricting) and SSGS. All correlations are significant at *p* <.05, with 95% confidence intervals shown

### Correlation between SSGS and EPSI subscales

A Pearson correlational analysis was conducted to examine the relationships between the SSGS and the subscales of the EPSI. This analysis revealed several significant associations. Most notably, SSGS demonstrated a strong positive correlation with body dissatisfaction (*r* =.357, *p* <.001), restrictions (*r* =.287, *p* <.001), and purging (*r* =.202, *p* =.021). No other significant correlations were observed with the remaining EPSI subscales (binge eating, cognitive restraint, excessive exercise, muscle building, and negative attitudes toward obesity) (Fig. 4).

## Discussion

The present study aimed to explore factors associated with EPS in young adult athletes. The first research question examined whether demographic variables and moral emotions of shame and guilt significantly predict EPS. The second research question investigated whether shame and guilt mediate the relationship between the type of sport and EPS. The third research question explored which subscales of EPS are most strongly correlated with the total score of shame and guilt.

In line with the first research question, hierarchical regression analysis revealed that both the type of sport (weight-sensitive vs. non-weight-sensitive) and momentary experiences of shame and guilt (measured by the SSGS) were significant predictors of EPS. In contrast, other demographic variables, including age, gender, living arrangements, and membership duration, did not significantly predict EPS.

Specifically, athletes participating in weight-sensitive sports reported significantly higher levels of EPS than those involved in non-weight-sensitive sports did. This aligns with prior literature identifying weight-sensitive sports as a prominent risk factor for disordered eating behaviors [34, 35]. These findings underscore the influence of sport-specific pressures, suggesting that athletes may develop EPS regardless of their emotional state if their sporting environment strongly emphasizes weight or appearance. Conversely, participation in non-weight-sensitive sports may have a protective effect, potentially due to a reduced emphasis on body weight and shape. This interpretation is consistent with previous research indicating that less weight-focused environments can buffer against body dissatisfaction and other EPS [9, 10, 41]. These results have practical implications for prevention efforts. Interventions targeting EPS in athletes may benefit from addressing sport-specific norms and coaching practices rather than focusing solely on individual emotional factors. Promoting body-positive messaging, fostering supportive team cultures, and offering training to coaches on how to avoid reinforcing weight-related pressures could help mitigate risk. Additionally, early screening for EPS in weight-sensitive sports might be needed, even among athletes who do not demonstrate high levels of shame or guilt.

Although the effects of shame and guilt are modest, they are both statistically significant and conceptually important. The association between higher levels of state shame and guilt and greater eating pathology suggests that these self-conscious emotions may act as risk factors for disordered eating, in line with previous research [26, 32]. While earlier studies indicated that guilt may have a weaker association with eating disorder pathology [6], our results revealed a notable relationship between guilt and EPS. This finding supports the broader notion that negative self-evaluative emotions contribute meaningfully to EPS.

These findings have several implications from a clinical perspective. First, interventions for athletes at risk of disordered eating may benefit from incorporating strategies to identify and regulate moral emotions of shame and guilt, particularly in the context of sport-related performance or appearance pressures. Techniques drawn from compassion-focused therapy or emotion regulation training may offer promising avenues for helping athletes respond to these emotions more adaptively and avoid developing moral injury and clinical EPS [50]. Second, routine screening for emotional distress within sport settings may help identify early signs of vulnerability, even among athletes without prior mental health diagnoses. In particular, further exploration of the effects of moral emotions such as shame, guilt, and self-blame, as well as the concept of moral injury, is needed. These experiences may represent underrecognized clinical concerns that align with or exacerbate the pathology of eating disorders. Understanding how moral emotions and further moral injury contribute to the development and maintenance of EPS could enhance early intervention strategies and inform more tailored psychological support for athletes. Finally, this study highlights the importance of considering both environmental aspects, including sport type and culture, and intrapersonal aspects, represented by moral emotions, in understanding EPS. The interaction between sport-specific pressures and internal emotional experiences suggests a dynamic interplay that requires further exploration, including longitudinal designs to assess the potential cumulative impact over time.

Unexpectedly, demographic variables such as gender, age, living arrangements, and membership duration did not significantly predict EPS. The nonsignificant effect of gender is surprising given the extensive literature linking female gender with higher levels of disordered eating [10, 19, 27]. However, recent work highlighting eating disorder pathology in male athletes, particularly in relation to muscularity ideals [21, 31], may explain the diminished gender difference observed here. Similarly, the narrow age range of the participants (18–25 years) may have obscured potential age-related differences. Despite this age group being a known period of elevated eating disorder pathology risk [33], the homogeneity of the sample likely limited the variability required to detect meaningful effects.

According to the second research question, athletes participating in weight-sensitive sports reported significantly higher levels of eating disorder pathology; however, this relationship appeared to be direct rather than mediated by state shame or guilt. This finding differs from several previous studies that positioned shame, particularly body-related shame, as a central emotional mechanism underlying eating disorder pathology [26, 32], as it was expected that athletes in weight-sensitive sports would internalize idealized body standards and experience shame or guilt when failing to meet those ideals [5, 13]. Our results suggest that other psychological or environmental factors may play a more central role in shaping vulnerability to disordered eating in these sports. For example, trait and socially prescribed perfectionism may drive athletes to rigidly pursue idealized body standards, while coach-related appearance pressures and performance feedback may further reinforce disordered eating behaviors [3, 9, 18, 23, 25, 26, 29, 36, 43]. Together, these factors could operate independently of shame or guilt, highlighting the complex interplay of individual and environmental influences in weight-sensitive athletic contexts.

Future research would benefit from modeling multiple mediators simultaneously to better capture these intersecting influences, particularly through longitudinal assessments that can track the dynamic interplay between emotional states, personality traits, and environmental stressors over time.

According to the third research question, the results suggest that shame and guilt, as measured by the SSGS, is particularly associated with attitudinal and compensatory aspects of eating pathology, including body dissatisfaction, restrictive eating, and purging, rather than with other behavioral or cognitive components. This finding is consistent with theoretical models that highlight the role of shame and guilt in driving body-focused and compensatory eating behaviors, underscoring their relevance for understanding specific dimensions of disordered eating in athletes [9, 27, 37]. Future research could examine these associations longitudinally to better understand the potentially causal relationships between self-conscious negative affect and specific eating pathology dimensions.

## Strengths, limitations, and future directions

A main feature of this study lies in its focus on subclinical EPS in a nonclinical population of university athletes, a group often overlooked in eating disorder pathology research. By targeting university athletes, the present study contributes to the growing body of research advocating for early detection and intervention to prevent progression from subclinical to clinical eating disorders. While this decision allowed us to focus on subclinical manifestations of eating disorder pathology and examine early risk and protective factors, it also limits the generalisability of the findings to athletes with clinically diagnosed eating disorders. Future research should consider including both clinical and nonclinical populations to better understand the full spectrum of eating disorder pathologies and how factors such as shame, guilt, and sport type may function differently across diagnostic thresholds.

Other limitations of the current study should also be acknowledged. First, recruitment challenges such as low response rates from university sport societies may have introduced self-selection bias. Athletes with strong emotional responses or concerns about body image may have chosen not to participate because of questionnaire sensitivity, stigma, or time constraints [49]. Second, reliance on self-reported data introduces potential biases, such as social desirability or underreporting of sensitive behaviors. Within athletic settings, where emotional vulnerability is often stigmatized [45], participants may have downplayed shame, guilt, or disordered eating. The incorporation of mixed-method approaches, such as interviews or observational data, would enhance validity in future studies.

Although shame and guilt were significant predictors of EPS, their effects were modest and should be interpreted with caution; importantly, they did not mediate the relationship between sport type and EPS. Future studies should examine a broader range of protective factors. Educating coaches and sports professionals on how to recognize and appropriately respond to eating disorder pathology is important, given their influential role in athletes’ lives.

## Conclusion

This study examined predictors of EPS in young adult athletes, focusing on the type of sport, shame and guilt, and demographic factors. Weight-sensitive sports and higher levels of shame and guilt were significantly associated with greater EPS. In contrast, participation in non-weight-sensitive sports may serve as a protective factor against disordered eating. This highlights the need to explore alternative psychological and contextual mediators, particularly in relation to specific forms of pathology such as body dissatisfaction, restrictive eating behaviors, and purging, which are typically closely linked to moral emotions of shame and guilt.

Future research should employ longitudinal studies and mixed-method designs to examine potential risk and protective factors against EPS, including perfectionism and coaching pressure. Overall, the findings contribute to our understanding of the pathology of eating disorders in university athletes and emphasize the importance of early detection and protective interventions in sport environments.

## Data Availability

The analysis code is available at https://github.com/LZasiekina/Eating-Pathology-Symptoms.git.
